# CORUM: the comprehensive resource of mammalian protein complexes–2022

**DOI:** 10.1093/nar/gkac1015

**Published:** 2022-11-16

**Authors:** George Tsitsiridis, Ralph Steinkamp, Madalina Giurgiu, Barbara Brauner, Gisela Fobo, Goar Frishman, Corinna Montrone, Andreas Ruepp

**Affiliations:** Institute of Experimental Genetics, Helmholtz Center Munich (GmbH), German research Center for environmental Health, Neuherberg D-85764, Germany; Institute of Experimental Genetics, Helmholtz Center Munich (GmbH), German research Center for environmental Health, Neuherberg D-85764, Germany; Experimental and Clinical Research Center, Max Delbrück Center for Molecular Medicine and Charité Universitätsmedizin Berlin, Berlin 13125, Germany; Institute of Experimental Genetics, Helmholtz Center Munich (GmbH), German research Center for environmental Health, Neuherberg D-85764, Germany; Institute of Experimental Genetics, Helmholtz Center Munich (GmbH), German research Center for environmental Health, Neuherberg D-85764, Germany; Institute of Experimental Genetics, Helmholtz Center Munich (GmbH), German research Center for environmental Health, Neuherberg D-85764, Germany; Institute of Experimental Genetics, Helmholtz Center Munich (GmbH), German research Center for environmental Health, Neuherberg D-85764, Germany; Institute of Experimental Genetics, Helmholtz Center Munich (GmbH), German research Center for environmental Health, Neuherberg D-85764, Germany

## Abstract

The CORUM database has been providing comprehensive reference information about experimentally characterized, mammalian protein complexes and their associated biological and biomedical properties since 2007. Given that most catalytic and regulatory functions of the cell are carried out by protein complexes, their composition and characterization is of greatest importance in basic and disease biology. The new CORUM 4.0 release encompasses 5204 protein complexes offering the largest and most comprehensive publicly available dataset of manually curated mammalian protein complexes. The CORUM dataset is built from 5299 different genes, representing 26% of the protein coding genes in humans. Complex information from 3354 scientific articles is mainly obtained from human (70%), mouse (16%) and rat (9%) cells and tissues. Recent curation work includes sets of protein complexes, Functional Complex Groups, that offer comprehensive collections of published data in specific biological processes and molecular functions. In addition, a new graphical analysis tool was implemented that displays co-expression data from the subunits of protein complexes. CORUM is freely accessible at http://mips.helmholtz-muenchen.de/corum/.

## INTRODUCTION

Cellular systems in mammals can be viewed as the product of ∼20 000 individual protein-coding genes acting in concert to carry out catalytic, structural and regulatory functions. Coordination is orchestrated through protein-protein interaction networks that assemble functionally related gene products into structures such as protein complexes and organelles. Therefore, it is of central interest towards a better understanding genotype-phenotype relationships to obtain knowledge about all protein complexes in living cells.

A first high-throughput approach in an eukaryotic organism was performed in Saccharomyces cerevisiae and defined 232 distinct multiprotein complexes (1). Recent proteomic experiments discovered a human protein complex map consisting of 6965 different complexes (2). Other approaches are tackling the complete human interactome (3). All these large-scale affinity-purification mass spectrometry analyses require benchmark datasets for evaluation.

In previous decades, thousands of protein complexes from mammalian organisms have been characterized in individual experiments with respect to subunit composition and cellular function. The CORUM database offers the largest publicly available compendium of manually curated mammalian protein complexes based on experimental results from the literature (4). It served as resource and benchmark for all above mentioned major endeavors for the characterization of the human interactome and proteome.

With the new release CORUM 4.0 we further extended the dataset to 5204 protein complexes. In the first releases our aim was to cover a broad spectrum of protein complexes from different areas. A representative set of complexes is a requirement to serve as reference dataset for large-scale approaches for investigating the mammalian complexome, for the development of data analysis tools (5) and for analysis of experimental data from different kinds of diseases such as cancer (6). With the new CORUM 4.0 release we also provide sets of protein complexes, Functional Complex Groups (FCGs), that offer comprehensive collections of published data in specific biological processes and molecular functions. In addition, a graphical analysis tool was implemented that displays co-expression data from the subunits of protein complexes. The tool is based on the Cytoscape javascript (7), an open-source library for visualizing complex networks, and uses data from the STRING server. CORUM is freely accessible at http://mips.helmholtz-muenchen.de/corum/.

## RESULTS AND DISCUSSION

### Extended content and application of CORUM

**Figure 1.**
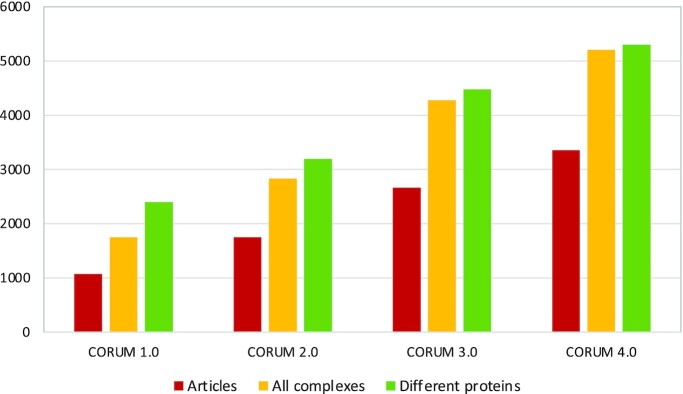
Data growth in CORUM. The plot compares the data content of CORUM versions 1.0, 2.0, 3.0 and 4.0. It includes the number of articles that were used to create the datasets, the total number of protein complexes as well as the total number of different proteins that are found in the dataset.

**Figure 2.**
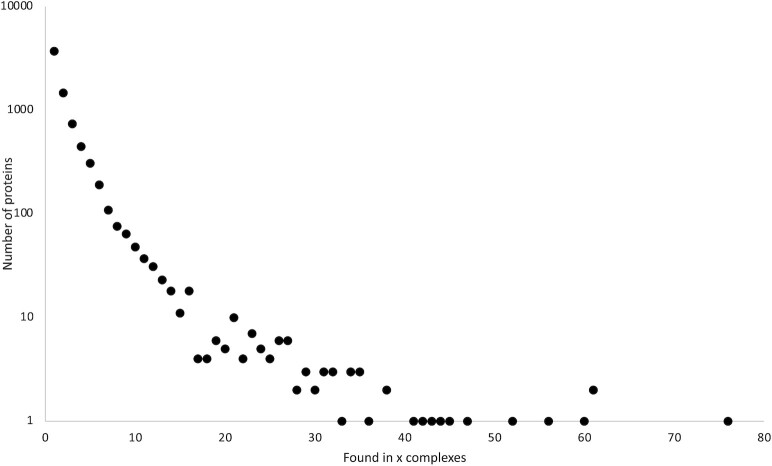
Reuse of proteins in mammalian protein complexes. The number of proteins (y-axis) participating in a particular number of complexes (x-axis) is plotted. There are for example 740 proteins, which are subunits in three protein complexes in the CORUM 4.0 dataset.

Compared with the CORUM 3.0 release (4) the number of protein complexes was further increased from 4274 to 5204 (Figure 1). One particular goal was to cover the mammalian proteome more extensively. As a result, the CORUM 4.0 release now includes 5299 different proteins representing ca. 26% of human protein coding genes (19.969) (8). Analysis of the yeast interactome network showed that subunits of protein complexes are likely to be essential (9). In the CORUM dataset, a substantial proportion of proteins (30%) are found as subunit of more than one protein complex. Proteins such as Integrin beta-1 and histone deacetylase 1 are subunits in as many as 60 and 76 protein complexes, respectively (Figure 2). Reutilisation of subunits is an extensively used feature in mammalian cells to increase the functionality of individual proteins. The mitotic checkpoint protein BUB3 for example, is not only involved in chromosome segregation (Mitotic checkpoint complex; complex-ID 190), but is also part of the spliceosome A complex (complex-ID 8372) which is involved in RNA processing. With the curation of more protein complexes and progress in human complexome research the number of multiply used complex subunits will increase significantly. Compared with the CORUM 3.0 release, the number of proteins found in at least two different complexes increased from 2630 to 3250 in CORUM 4.0 and the fraction of reused proteins increased from 59% to 61%, respectively.

Another option of cells providing protein complexes with different functions is the utilisation of isoforms (splice variants). Driven by progress in mass spectrometry technology there is an increasing number of publications where particular isoforms of complex subunits were identified. Hence, we are now also annotating isoform information in CORUM if the respective information is available (e.g. complex 7590: CASP2(S isoform)-SPTAN1 complex). Similar to isoforms, we also provide information about post translational processing (see complex ID 7529).

In order to streamline to content of the dataset we removed protein complexes with identical subunit composition from the same organism which have been characterized in more than one publication or by different methods. As a consequence, we do not offer a core set any more. Compared with the previous release, the distribution of organisms as source of protein complex analysis has not changed considerably. The vast majority of complexes was characterized in human cells with 70% (3637) followed by 16% mouse (847) 9% rat (461).

An important application of the CORUM dataset is disease research. About one third of citations from the latest CORUM article is dedicated to disease research. These include various applications such as databases (10), tools that develop clinical biomarkers (6), or the analysis of experimental data (11). The largest fraction of covered diseases is cancer, some applications with a broad coverage of cancer types (6), others analyzed data from particular cancer types such as head and neck cancer (12) or ovarian cancer (13). Moreover, the CORUM dataset is also used for infectious diseases such as the recent COVID-19 pandemic. Here, protein complex information is being used in landmark publications to discover host-pathogen interactions and to reveal targets for drug repurposing (14–16).

Diseases are often caused not by dysfunction of individual proteins but by deregulated protein complex function. Examples are the spliceosome in cancer (10) and the proteasome in neurodegenerative diseases (17). OMIM is a resource offering extensive information on proteins causing inherited diseases (18). Since results in Mycoplasma and yeast found that almost 90% of soluble proteins were part of at least one complex (19) it is tempting to speculate that in many instances the etiology of human diseases is associated with dysfunctional protein complexes. In order to present information about the potential influence of subunits on diseases, we linked OMIM information about associated diseases with all proteins in the CORUM dataset where respective information was found. This information offers the opportunity to predict disease-associated protein complexes. In the ‘Intraflagellar transport complex B peripheral subcomplex’ (complex-ID 6174) for example, mutations in four out of six subunits were found to cause skeletal anomalies. For the two subunits CLUAP1 and IFT20, so far no diseases are reported in OMIM. If in children with skeletal development defects no mutations are found in the genes known for this phenotype, causative mutations might be found in CLUAP1 or IFT20. Results from the literature support this hypothesis: IFT20 is involved in the craniofacial bone formation in mouse (20) and in a case study it was found that biallelic mutations in CLUAP1 cause craniofacial anomalies (21).

In addition to various kinds of data analyses, CORUM became a broadly used information resource which is applied in more than 50 biomedical databases and research tools (Supplementary Table S1). Beside CORUM, few other sources offer curated information on protein complexes in mammals. Respective data can be found in the ‘cellular component’ section of the Gene Ontology (22), as part of molecular pathways in the Reactome database (23) or in the Protein Complex Portal (24). The Complex Portal also offers protein complex information for other organisms than mammals. Different to the Complex Portal, CORUM does not transfer information between organisms but only offers data from the organismal resource that has been analysed in respective publications. Another distinction between the two resources is that CORUM does not annotate non-protein components from protein complexes.

### Description of functional complex groups

**Figure 3.**
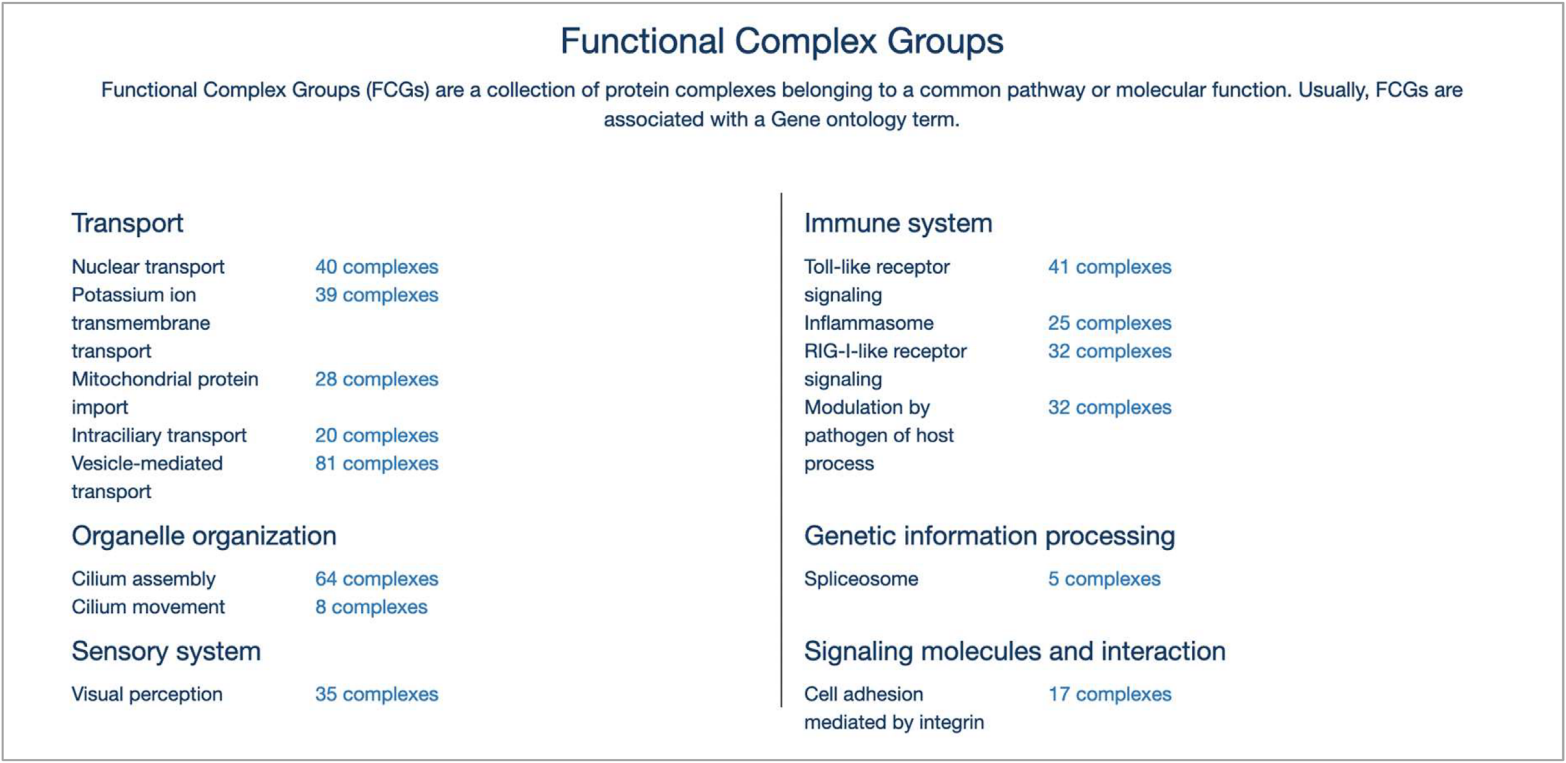
Dataset of functional complex groups (FCGs) in CORUM. The homepage shows available FCGs and the respective number of members.

As shown above, there are multiple experimental and bioinformatics approaches trying to achieve a complete representation of the cellular machinery. To obtain an estimate about the completeness of the results requires reference datasets which are comprehensive, at least based on current knowledge. Also, for the analysis of high-throughput data with a medical context it is valuable information to know if a biologically meaningful group of complexes is overrepresented in a disease condition. Hence, we started to annotate groups of protein complexes belonging to a biological process or the same molecular function (Figure 3). The compilation of these groups, so called functional complex groups (FCGs), is oriented on terms from the Gene Ontology (22).

The FCG ‘Potassium ion transmembrane transport’ for example covers a group of protein complexes playing important roles in vital cellular signalling processes in both excitable and non-excitable cells. Naturally occurring mutations in various K(+) channels cause cardiovascular, neurological or metabolic diseases (25). In the field of cardiac arrhythmias for example there are considerable efforts to modify potassium channel activity with chemical compounds such as PUFA analogs (26).

Most of the mitochondrial proteins are encoded by nuclear DNA and their precursors have to be transported into the mitochondria. The FCG ‘Mitochondrial protein import’ describes the set of complexes that perform this process. Pathological effects of e.g. TOM (mitochondrial outer membrane) and TIM (mitochondrial inner membrane) complex dysfunctions affect the protein import into mitochondria and are associated with Alzheimer's disease where it results in an inhibition of respiratory complexes and finally in an increased level of reactive oxygen species (27).

Non-motile (primary) cilia are sensory organelles, that transduce signals from the environment or from other cells, motile cilia move extracellular fluids like cerebrospinal fluid or propel sperm cells. The FCG ‘Cilium assembly’ comprises complexes like the CPLANE complex, the intraflagellar transport complexes IFT-A and IFT-B, or the BBSome (28) which interact in the generation of cilia. As cilia are found in most vertebrate cell types, there is a wide range of clinical features associated with disorders caused by alteration of cilia structure or function, the so called ciliopathies (28). Mutations in the subunits of the CPLANE, IFT-A, IFT-B and BBSome complexes can cause diseases like Bardet-Biedl syndrome, Short-rib thoracic dysplasia, Retinitis pigmentosa, Nephronophthisis or Spermatogenic failure, which reflect the broad spectrum of phenotypes associated with ciliopathies.

For the investigation of various biological systems, the scientific community established the use of particular mammalian model organisms, for example rat for potassium ion transporters. However, as no organism covers all known protein complexes in a field and we aim to be as complete as possible, FCGs are a collection of complexes from different organisms. If a protein complex has been characterized in different organisms, we preferably use representatives from human cells or tissues. FCGs and the number of representatives are shown on the homepage (Figure 3) and can be viewed via hyperlink. In addition, FCGs are available as download in table format.

### Co-expression of protein complex subunits

Analyses of eukaryotic protein complexes revealed that complexes are comprised of a core in which subunits are highly co-expressed and represent functional units (1,29). This core is surrounded by groups of proteins acting as modifiers of the complex's function or other, functionally unrelated proteins that spuriously attach to the surface of the core proteins. The core is expected to remain stable under different conditions, whereas significant changes may occur in attached proteins.

In order to provide an insight into the organization of protein complexes, and to provide a perspective on the role of the various protein subunits, the new CORUM release presents a graphical tool that integrates known protein co-expression information. A large publicly available collection of co-expression data is provided by the STRING database (30). STRING offers an application programming interface (API) which enables programmatic access of the co-expression data. CORUM retrieves the co-expression between protein complex subunits using the STRING network API method. For visualization of co-expression between protein complex subunits we use Cytoscape (7). Cytoscape.js is a javascript-based graph-visualization library that we embedded in version 3.2 on our website. The color of each edge represents the co-expression score using a color gradient. The tool allows to set individual thresholds for co-expression.

**Figure 4.**
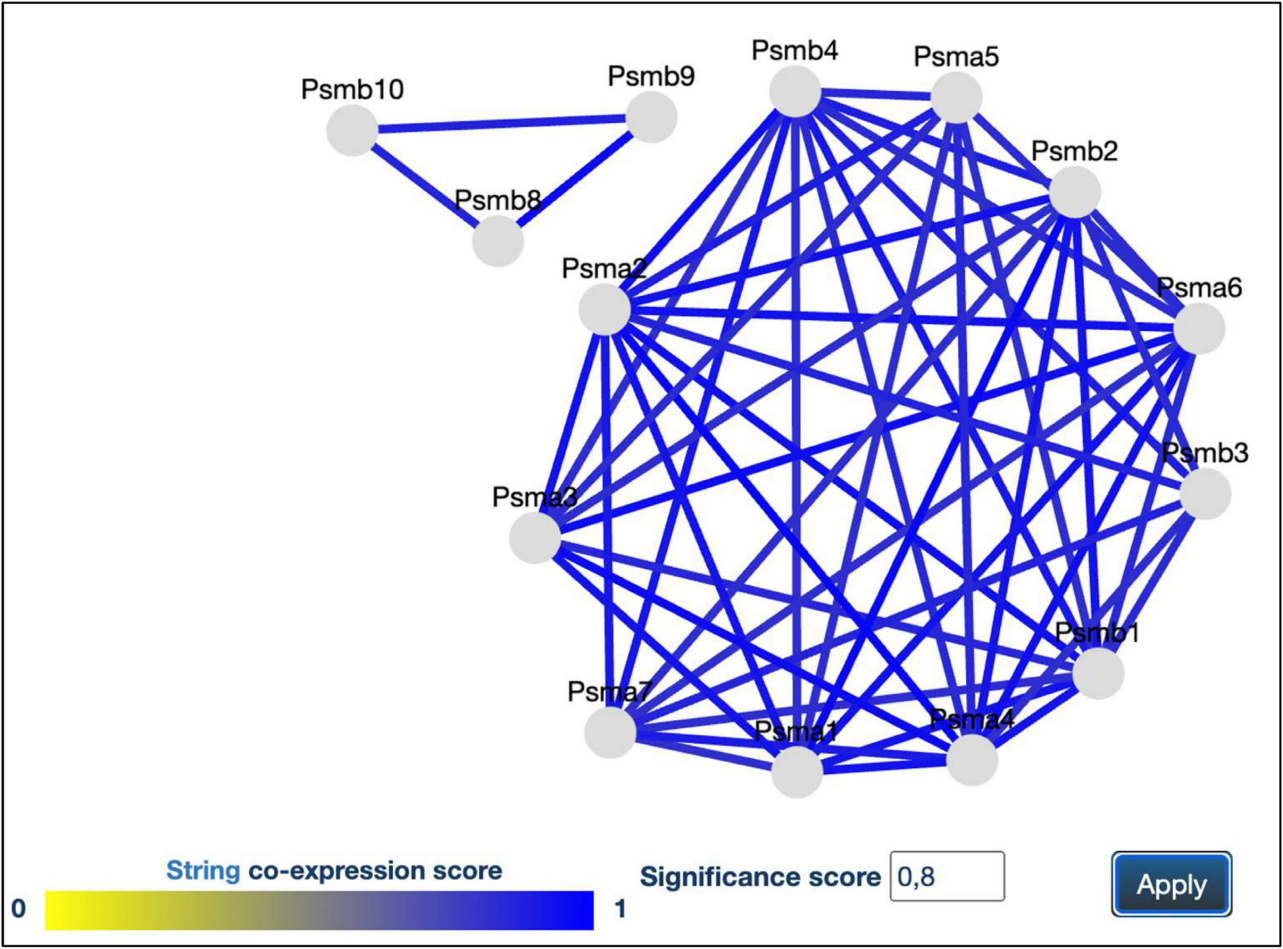
Co-expression of the immunoproteasome subunits shown on the CORUM interface for gene co-expression. At a significance score of 0.8, subunits which are specific for the immunoproteasome form a cluster that is separated from subunits of the constitutive proteasome. All protein complex entries where the respective information is available, are linked to a co-expression interface.

The application of the tools is shown for the proteasome (Figure 4), a molecular machine that catalyzes the degradation of most cellular proteins (31). When cells in liver and other tissues of vertebrates are stimulated with pro-inflammatory cytokines, most of their constitutively expressed proteasomes are replaced with immunoproteasomes. These contain three additional β1i (PSMB9), β2i (PSMB10) and β5i (PSMB8) subunits which are preferentially incorporated into proteasomes instead of subunits β1, β2 and β5. Compared with the constitutive proteasome, the immunoproteasome has a different proteolytic activity, which increases the production of peptides for presentation on MHC class I molecules (31). With respect to co-expression the three immunoproteasome-specific subunits form a cluster which is separated from the core proteasome (Figure 4).

## CONCLUSIONS AND FUTURE DEVELOPMENTS

In recent years, CORUM became an important resource in the fields of biology and biomedicine, by collecting experimentally characterized protein complexes to create a reference dataset for data analysis and bioinformatics applications. Expert curation is a key focus of our activities, to inform on composition, functional role and other features of protein complexes. The CORUM dataset continues to grow and to incorporate more complexes in particular from human tissues and cells. Recent improvements were driven by our efforts not only to increase the amount of protein complexes (5204) but also the coverage of the mammalian proteome (26%).

The growing importance and application of protein complex information in disease research is demonstrated by the high number of publications that made use of the CORUM 3.0 dataset for the analysis of disease-associated data or the creation of biomedical analysis tools. In particular worth mentioning is pioneering research for the understanding of molecular processes in COVID-19. To provide the CORUM dataset with additional disease-related information, the complex subunits are now linked to respective OMIM diseases.

With CORUM 4.0 we have begun to offer Functional Complex Groups. These represent comprehensive sets of protein complexes with common biological or biomedical relevance (molecular function or bioprocess). For projects aiming to represent a complete mammalian complexome, FCGs will give hints to what extent this goal has been accomplished. Also, for the analysis of biomedical data, it may offer valuable insight if representatives of FGCs are overrepresented.

Future plans include (i) the addition of further FCGs with a focus on biomedical relevance, (ii) to serve the community with an expanding the set of tools for exploring protein complex information, (iii) to enable the prediction of impacts of deregulated protein complex function in the context of disease symptoms and (iv) to integrate structural information such as data from cryo-electron microscopy.

We invite all researchers to send us feedback and suggestions for incorporation of additional protein complexes from the user community. Please contact us at andreas.ruepp@helmholtz-muenchen.de.

## DATA AVAILABILITY

The provided data can be freely downloaded in various formats from our downloads page (http://mips.helmholtz-muenchen.de/corum/#download) under the Creative Commons Attribution License (CC BY 4.0).

## Supplementary Material

gkac1015_Supplemental_File
